# Loss-of-Function Mutations in Rab Escort Protein 1 (REP-1) Affect Intracellular Transport in Fibroblasts and Monocytes of Choroideremia Patients

**DOI:** 10.1371/journal.pone.0008402

**Published:** 2009-12-22

**Authors:** Natalia V. Strunnikova, Jennifer Barb, Yuri V. Sergeev, Ashwin Thiagarajasubramanian, Christopher Silvin, Peter J. Munson, Ian M. MacDonald

**Affiliations:** 1 Ophthalmic Genetics and Visual Function Branch, National Eye Institute, National Institutes of Health, Bethesda, Maryland, United States of America; 2 Mathematical and Statistical Computing Laboratory, Center for Information Technology, National Institutes of Health, Bethesda, Maryland, United States of America; 3 Genetics and Molecular Biology Branch, National Human Genome Research Institute, National Institutes of Health, Bethesda, Maryland, United States of America; 4 Department of Ophthalmology, University of Alberta, Edmonton, Alberta, Canada; Instituto Gulbenkian de Ciência, Portugal

## Abstract

**Background:**

Choroideremia (CHM) is a progressive X-linked retinopathy caused by mutations in the *CHM* gene, which encodes Rab escort protein-1 (REP-1), an escort protein involved in the prenylation of Rabs. Under-prenylation of certain Rabs, as a result of loss of function mutations in REP-1, could affect vesicular trafficking, exocytosis and secretion in peripheral cells of CHM patients.

**Methodology/Principal Findings:**

To evaluate this hypothesis, intracellular vesicle transport, lysosomal acidification and rates of proteolytic degradation were studied in monocytes (CD14+ fraction) and primary skin fibroblasts from the nine age-matched controls and thirteen CHM patients carrying 10 different loss-of-function mutations. With the use of pHrodo™ BioParticles® conjugated with *E. coli*, collagen I coated FluoSpheres beads and fluorescent DQ™ ovalbumin with BODYPY FL dye, we demonstrated for the first time that lysosomal pH was increased in monocytes of CHM patients and, as a consequence, the rates of proteolytic degradation were slowed. Microarray analysis of gene expression revealed that some genes involved in the immune response, small GTPase regulation, transcription, cell adhesion and the regulation of exocytosis were significantly up and down regulated in cells from CHM patients compared to controls. Finally, CHM fibroblasts secreted significantly lower levels of cytokine/growth factors such as macrophage chemoattractant protein-1 (MCP-1), pigment epithelial derived factor (PEDF), tumor necrosis factor (TNF) alpha, fibroblast growth factor (FGF) beta and interleukin (lL)-8.

**Conclusions/Significance:**

We demonstrated for the first time that peripheral cells of CHM patients had increased pH levels in lysosomes, reduced rates of proteolytic degradation and altered secretion of cytokines. Peripheral cells from CHM patients expose characteristics that were not previously recognized and could used as an alternative models to study the effects of different mutations in the REP-1 gene on mechanism of CHM development in human population.

## Introduction

Choroideremia (CHM) is an X-linked monogenic disease caused by various mutations in the *CHM* gene that result in the loss of function of Rab escort protein (REP-1) and cause slow degeneration of the retinal pigment epithelium (RPE), choroid and photoreceptors. By the age of 40, a male patient with CHM will typically have a significantly limited peripheral visual field, usually at the level of legal blindness (less than 20 degrees)(**Table 1**). Mutations in the *CHM* gene include full deletions, partial deletions (intragenic and other), deletion/insertions, splice site mutations and nonsense mutations [1], [2], [3], [4]. Immunoblot analysis of protein from white blood cells of CHM patients shows that most patients lack REP-1 [5]. REP-1 is involved in post-translational lipid modification (isoprenylation) of monomeric Rab GTPases (Rabs), which are key regulators of vesicular trafficking, phagosome fusion and maturation [1], [6], [7]. Seabra and colleagues first showed that Rab27 was unprenylated in lymphoblasts of CHM patients and was more efficiently prenylated by REP-1 than REP-2 [8]. Recent work in *C. elegans* has demonstrated that rep-1 may prenylate specific Rabs in specific tissues, such as rab-27 which is involved in synaptic transmission, and does not participate in the prenylation of other rabs [9]. Mammals have a *CHM* gene and a *CHM-like* gene that encodes REP-2, which is thought to partially compensate for the lack of REP-1 in all tissues except the eye in CHM patients [10].

**Table 1:** Clinical characteristics of CHM patients and expected effect of determined mutations on the structure of REP-1 protein.

Patient ID, Age (y.o.)	Visual Acuity*	Visual Field (III4e)	Mutation and expected molecular mass	Expected structural change
CHM 1 29	OD:20/20 OS:20/20	Central 20°, with peripheral remnants (OU)	**p.I460X**; exon 10, c.1327_1328delA; 52 kDa	REP-1 sequence ends at N442 followed by the peptide VLTCAIQADLQGSADYRI at the C-terminus.
CHM 2 61	OD:20/20 OS:20/80	Central 2° (OU)	Full deletion; 0 kDa	No protein product.
CHM 3 39	OD:20/25 OS:20/40	Central 5° (OU)	**p.Q273X**; exon 6, Q273X;c.817C>T; 30 kDa	Protein chain is truncated after residue Q273.
CHM 4 49	OD:20/40-3 OS:20/63	Central 3° (OU) with temporal (OD), inferior (OS) islands	**p. I553X**; exon 14, c.1646delC; 63 kDa	REP-1 sequence ends at W548 followed by the peptide VFTSI at the C-terminus†.
CHM 5 54	OD:20/640 OS:20/32	15° (OD) 10° (OS)	**p. I244X**; deletion exons 6+7; 27 kDa	REP-1 sequence ends at K234 followed by the peptide DMKRSHFMNI at the C-terminus.
CHM 6.1** 18	OD:20/20 OS:20/20	Central 12° (OU)	**p.K234X**; exon 5, c.700A>T; 26 kDa	Protein chain is truncated after residue K234. All structural domains are affected by this change.
CHM 6.2** 16	OD:20/20 OS:20/16	140° (OD) 150° (OS)	**p.K234X**; exon 5, c.700A>T; 26 kDa	Protein chain is truncated after residue K234.
CHM 6.3** 13	OD:20/20 OS:20/16	120°, with 25° scotoma (OD); 140° with 20° scotoma (OS)	**p.K234X**; exon 5, c.700A>T; 26 kDa	Protein chain is truncated after residue K234.
CHM 7 54	OD:20/250 OS:HM	Central 1° (OD)	**p.M1I**; exon 1, c.3G>A; 57 kDa	Protein synthesis could start from the first Met in sequence (M149), resulting in a protein of 505 residues.
CHM 8 44	OD:20/160 OS:HM	Declined testing	**p.L550P**; exon 14, c.1679T>C, 73 kDa	Missense mutation changing the conformation and protein stability by breaking the hydrogen-bonding pattern.
CHM 9 74	OD:LP OS:NLP	Not recordable	**p.Y504X**; exon 13 c.1542T>A,STOP;57 kDa	Protein chain is truncated after residue Y504†.
CHM 10 22	OD:20/12 OS:20/16	140° (OU)	No mutation found; no copy number variants	n/a
CHM 11 46	OD:20/63 OS:20/25	10°(OD), 5° (OS) (V4e)	**p.P179X**; exon 5 c.525_526delAG; 20 kDa	Rep-1 sequence ends at the G175 followed by the peptide KRKP at the C-terminus (residues 176–179)†.

* HM, hand motion; LP, light perception; NLP, no light perception.

** Brothers carrying the same mutation in Rep-1 protein.

† effect of mutations I553X, L550P, Y504X and P179X previously analyzed by Sergeev et al. 2009.

Despite our knowledge of the molecular genetics of CHM and linked biochemical pathways, there is no clear understanding of its pathogenesis, the severity of the eye condition and its progression. Researchers have had few suitable animal models to study the pathogenesis of CHM. For example, a male mouse knock out model of CHM has not been produced. The female CHM carrier will not carry an affected male pup to term as the normal vasculogenesis of the placenta is altered [11]. Conditional knockouts of the *Chm* gene in photoreceptors and RPE of mice have been created and have confirmed cell-autonomous degeneration in these tissues [12]. A number of studies have suggested that mutations in REP-1 could cause under-prenylation of certain Rabs and influence trafficking and outer segment disc phagocytosis by RPE cells [13], [14], [15], [16]. Krock and colleagues [15] used the *chm* null zebrafish model to confirm that the absence of rep-1 affects phagocytosis and trafficking in RPE cells, clearly showing defects in the elimination of undigested outer segment disc membranes. They suggested that the lack of rep-1 alters the rab27a/myosin7a complex that in turn affects RPE phagocytosis [15].

Ideally, one would wish to study trafficking defects in the eyes of CHM patients; however, eye tissue from these patients is not likely to be available for experimentation. Human RPE cultures models would be hard to obtain from CHM patients, and further, culture and manipulate. Monocytes (CD14+ fraction) and primary skin fibroblasts from CHM patients offer research material to indirectly evaluate the effect of different mutations on the disease phenotype, as loss of function mutations in the *CHM* gene are present in every tissue of affected individuals. This approach has allowed us to overcome the limitations of animal models and directly evaluate the disease phenotype in different tissues. To our knowledge, this study is the first to look at the effect of different mutations in the *CHM* gene on phagocytosis, intracellular trafficking, proteolytic degradation and secretion in monocytes (CD14+ fraction) and primary skin fibroblasts of CHM patients carrying different mutations in the *CHM* gene. Intracellular vesicle transport and the rates of proteolytic degradation were evaluated in peripheral cells using pHrodo™ BioParticles® conjugated with *E.coli*, collagen I coated FluoSphere beads and fluorescent DQ™ ovalbumin with BODYPY FL dye. A significant increase in the levels of lysosomal acidification and decrease in the rates of proteolytic degradation was observed in monocytes from CHM patients compared to controls. In addition, primary fibroblasts from CHM patients exhibited significant differences in the uptake and trafficking of collagen-coated beads compared to controls.

Differences in the expression of genes involved in phagocytosis, secretion, and trafficking in peripheral cells from CHM patients could be correlated with the clinical presentation and progression of the disease. Under-prenylation of certain Rabs could impair exocytosis and secretion in affected cells [17], [18], [19], [20]. This hypothesis was supported by the analysis of the secretory profiles of fibroblasts derived from CHM subjects and age-matched control fibroblast cultures. CHM fibroblasts secreted significantly lower levels of macrophage chemoattractant protein (MCP-1), pigment epithelial derived factor (PEDF), tumor necrosis factor (TNF) alpha, fibroblast growth factor (FGF) beta and interleukin (IL)-8 into the media. These combined observations on peripheral cells from CHM patients expose differences not previously recognized and will provide models to study the effect of different mutations in the *CHM* gene that cause a retinal phenotype and help us to better understand the pathogenesis of this disorder.

## Methods

### Patient's Population and Study Design

**Figure 1:**
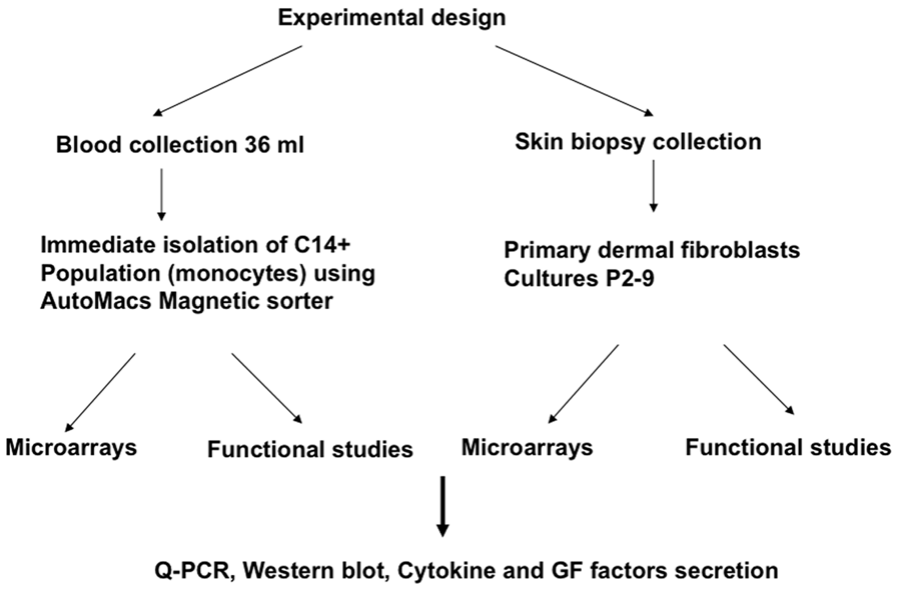
Experimental design. Collection of monocyte fractions and culture of primary dermal fibroblasts for the evaluation of gene expression and functional differences between CHM patients and age-matched controls.

This study was approved by the CNS Institutional Review Board of the NIH (08-E1-#017). A general outline of the experimental design is provided in **Fig. 1**. Informed written consent was obtained from each subject participating in this study. Thirteen males (age 13–74) with the clinical diagnosis of choroideremia were accrued through referral or self-referral to the National Eye Institute of the NIH, Bethesda, MD. An X-linked pattern of inheritance was either inferred or evident in all cases. Nine healthy males (age 27–78) with normal vision and no ocular pathology were recruited to act as age-matched controls. A complete ophthalmological examination was performed on all subjects with fundus photography and kinetic Goldmann visual field perimetry, where possible. For some patients, a full field ERG was recorded using Burian-Allen electrodes through dilated pupils according to ISCEV standards (http://www.ISCEV.org).

Peripheral blood samples (36 ml) were collected from CHM males and age-matched male controls, in a tube system containing sodium heparin and Ficoll Hypaque solution (Vacutainer CPT, Becton Dickinson, NJ). After immediate density gradient centrifugation, mononuclear cells were collected, washed, resuspended in 1 X PBS and separated using AutoMacs magnetic sorting to CD14+ and CD14- populations. Cells were then counted in a hemocytometer (Clay Adams Division of Becton Dickinson and Co., Parsippany, NJ) and fractions of the cells were used for phagocytic assays or frozen in Trizol (Invitrogen, Carlsbad, CA) for RNA extraction and microarray experiments.

### Primary Fibroblast Culture and Cell Viability after Drug Exposure

Primary dermal human fibroblasts cultures were used as a cellular model of CHM. With local anesthesia and a dermal punch, a skin biopsy was obtained from CHM affected patients and an age-matched healthy control group. The biopsy was immediately minced using sterilized forceps in a 60 mm dish containing 10% DMEM F12 media with 2.5 mg/l antibiotic/antimycotic (Invitrogen Life Sciences, Carlsbad, CA) and incubated for 10–14 days until fibroblasts started to attach to the plate. Media was changed every 2–3 days. Cells were spilt once they reached 80% confluence. As skin biopsies contain multiple cell types, immunocytochemistry with anti-collagen I antibody was performed on a subset of the primary culture to confirm the type of the cells used in the study. Cells at passage 2–7 were used for microarrays and functional studies. Cells from each patient were seeded in 96-well micro-culture plates at 100% confluence and oxidative injury was initiated by the treatment with 1, 10 and 50 uM of hydroquinone (HQ) in serum free media for 15 hours. Control cells were incubated with equal volume of serum free media. HQ was removed from the cells, replaced with regular media for 24 hours and after that the number of viable cells was determined by the addition of XTT- (sodium 3′-(phenylaminocarbonil)-3; 4-tetrasodium]-bis (4-methoxy-6-nitro) benzene sulfonic acid hydrate) using the manufacturer's instructions (Cell Proliferation Kit II; Roche Molecular Biochemicals, Indianapolis, IN).

### Molecular Genetic Testing

Genomic DNA was isolated from peripheral blood monocytes and then PCR-amplified using primer pairs as previously described [21] for analysis of all 15 coding exons of the *CHM* gene and their flanking splice sites. Bi-directional DNA sequence was obtained analyzed and compared to the published gene sequence. Molecular genetic testing was undertaken through the National Ophthalmic Genotyping Network, eyeGENE™ of the National Eye Institute, NIH.

### Homology Modeling of Human REP-1

Homology modeling of mutations in human REP-1 was carried out in a similar fashion to that described by Sergeev et al. [22]. Briefly, modeling of human REP-1 was based on the 2.2 Å crystal structure of rat Rep-1 protein (PDB file: 1vg0) as the structural template (Abola et al. 1987). Primary sequences of human and rat Rep-1 were aligned by the method of Needleman and Wunsch [23] and incorporated in the program Look, version 3.5.2 [24] for 3-dimensional structure prediction. Full-length REP-1 and changes in protein structure corresponding to 5 novel gene mutations: c.1327_1328delAT (p. I460X), c.817C>T (p. Q273X), deletion of exons 6 and 7 (p. I244x), c.700A>T (p. K234X) and c.3G>A (p. M1I) were built by the automatic segment matching method in the Look program followed by 500 cycles of energy minimization. In the full-length structure of REP-1, the elements of structure corresponding to the unresolved fragments of Rep-1 were generated in a conformation which has not been justified experimentally in order to show the possible location and structural role of these fragments in REP-1.

### Levels of Cytokines and Growth Factors in Conditioned Media

Levels of different cytokine/chemokines and growth factors: IL8, MCP-1, TNF alpha, epidermal growth factor (EGF), fibroblast growth factor (FGF), vascular endothelial growth factor (VEGF), and PEDF were measured in aliquots of conditioned media collected from primary fibroblasts of CHM and age matched control patients by commercial technology (SearchLight, Aushon BioSystems, Inc, Woburb, MA). To collect conditioned media, cells were grown to 100% confluence, and a week later regular media was substituted for a low serum (3%) media for 72 hours. After that, supernatant was collected, filtered, concentrated 2X using Centriprep® filter device (Millipore) and stored at −80°C.

### Phagocytic Assay

5×10^5^ primary fibroblasts from CHM and control patients were seeded onto each well of 6-well plates two days before the phagocytic assay (Falcon, Becton Dickinson, N.J., USA) in appropriate media containing 10% fetal calf serum. The monocyte fraction was used for the phagocytic assay after magnetic separation and plated either on 6 mm plates (10^6^ cells per well) or on 8 well chamber slides (5×10^5^ per chamber) (Lab-Tek; Nalge Nunc International, Naperville, IL) for live cell imaging. Phagocytosis in monocytes was tracked by pHrodo™ BioParticles® conjugate (pH dependent dye conjugated with *E. coli*, (Invitrogen, Carlsbad, CA)) and in fibroblasts with collagen-coated FluoSpheres (Invitrogen, Carlsbad, CA) using fluorescence-activated cell sorting (FACS) and live cell imaging analysis. On the day of the assay, regular media was replaced with SF Opti MEM media (Invitrogen, Carlsbad CA) and the number of BioParticles or FluoSpheres recommended by manufacturers was added to the cells. The bead concentration was not rate limiting. Stock solution of the pHrodo™ BioParticles® was 1 mg/ml and this suspension was diluted 1∶50 per sample including no-cell for background controls. The average fluorescence value of these no-cell background control wells was subtracted from all cell-containing wells at the end of the assay to determine a cell-specific, net phagocytosis signal. Cells were then incubated with the BioParticles for 30 min (monocytes) and 60 min with the FluoSpheres (fibroblasts) at 37°C in a humidified atmosphere containing 5% CO^2^. After that, the media containing fluorescent particles was removed and cells were washed twice with PBS and replaced with new SF Opti MEM. For the FACS analysis, primary fibroblasts were trypsinized immediately before analysis at each time point. Fluorescent and phase images of the cells were collected by the live imaging system from 4–5 different fields every 20–30 min over 12–15 h. To measure the effect of proton pump inhibitors on phagocytosis in CHM monocytes and fibroblasts compared to the controls, cells were pretreated for 30 min with 10 µM of bafilomycin A1 before the phagocytic assay. Following the treatment, cells were washed and BioParticles or FluoSpheres were added according to the protocol.

### Rate of Protein Degradation

Differences in the uptake and rate of degradation between the CHM patient and controls was determined using self-quenched conjugate of DQ™ ovalbumin with BODYPY FL dye, which exhibits green photostable and pH insensitive fluorescence upon proteolytic degradation (Molecular Probes, Eugene, OR). Ovalbumin conjugates are internalized via the mannose receptor–mediated endocytosis pathway and are recognized by antigen-presenting cells [25], [26]. The increase in fluorescence corresponding to the rate of degradation of engulfed DQ-ovalbumin was measured using FACS analysis at different time points following the feeding period.

### Confocal Microscopy

Fibroblasts and monocytes imaged with the confocal microscopy were plated on eight-well chamber slides 24 hours before the feeding (Lab-Tek; Nalge Nunc International, Naperville, IL). On the day of the assay, regular media was replaced with SF Opti MEM media (Invitrogen, Carlsbad CA) and the number of BioParticles or FluoSpheres recommended by manufacturers was added to the cells. Images of the cells were collected at different time points following the feeding. Confocal microscopy was performed on a laser scanning confocal microscope (model SP2, with TCS software version 11.04; Leica Microsystems, Exton, PA) using 40X and 63X objectives. Exciting with 351- and 364- nm laser beams and collecting emissions between 400 and 500 nm visualized staining with DAPI. Green fluorescence and AlexaFluor 488 staining was visualized by exciting with 488-nm laser beam and collecting emissions between 500 and 552 nm. pHrodo™ BioParticles® conjugate were visualized by exciting with a 568-nm laser beam and collecting emissions between 580 and 650 –nm. Scale bars are digitally included in some images.

### RNA Isolation and Expression Profiling

Fibroblasts and monocytes were collected, homogenized in TRIzol reagent (Invitrogen, Carlsbad, CA) and RNA was subsequently purified using an RNeasy Mini kit (Qiagen Inc, Valencia, CA). Double-stranded cDNA was synthesized from 6 µg of total RNA using the SuperScript Choice system (Invitrogen, Carlsbad, CA) and the T7-Oligo (dT) promoter primer kit (Affymetrix, Santa Clara, CA). cDNA was purified using Phase Lock Gels (Eppendorf, Westbury, NY). Biotin-labeled cRNA was then synthesized from double-stranded cDNA using the BioArray High Yield RNA transcript labeling kit (Enzo Diagnostics, Santa Clara, CA). cRNA was purified using an RNeasy Mini kit (Qiagen Inc, Valencia, CA) and fragmented into 35–200 base pair fragments by metal-induced hydrolysis (200 mM Tris-acetate, pH 8.2, 500 mM KOAc, 150 mM MgOAc) at 94°C for 35 minutes. Fifteen micrograms of fragmented biotin-labeled cRNA was hybridized onto Human Genome U133 Plus 2.0 GeneChips (Affymetrix, Santa Clara, CA) for 16 h at 45°C and 60 rpm. GeneChips were then washed and stained as per the Affymetrix GeneChip Expression Analysis Manual (Affymetrix, Santa Clara, CA). This procedure includes the removal of non-hybridized material. Staining was then performed with phycoerythrin-streptavidin and signal amplification by a second staining with biotinylated anti-streptavadin and phycoerythrin-streptavidin. Fluorescence from hybridized cRNA was detected using the Hewlett-Packard GS2500 Gene Array Scanner. Quality control measures included a requirement that scaling factors, percent of genes called present, average intensity, background values, housekeeping 3′/5′ ratios and measured intensities of spiked-in controls all fall within predefined ranges. Additionally, only samples with ‘acceptable’ amplification at the T7 RNA polymerase cRNA step were hybridized onto the chips.

### RT-PCR Validation Using TaqMan

Levels of mRNA in fibroblasts were confirmed by real time PCR using TaqMan one step RT-PCR and ABI Fast System 7900 (Applied Biosystems, Foster City, CA). Rt reactions were performed on 10 ug of total RNA and the total volume of the PCR reaction was 10 µL. The thermocycler parameters were 95°C for 10 min, followed by 40 cycles of 95°C for 15 s and 60°C for 1 min. Relative changes in gene expression were calculated using a variation of the ΔΔCt method. This method first subtracts from the gene threshold cycle (Ct) value, the average of the Ct values of 5 housekeeping genes (B2M, HPRT1, RPL13A, GAPDH, and ACTB), yielding a ΔCt value normalized to the RNA amounts for each of the four conditions studied. The ΔΔCt for each of the group was calculated by subtracting the mean ΔCt of the four groups from the ΔCt for each individual group. There were at least 3 biological replicates in each group.

### Protein Analysis

Fibroblasts, cultured adult RPE (ARPE19), human fetal RPE, human vascular endothelial cells (HUVEC), and peripheral blood mononuclear cells (PBMCs) were collected, washed and homogenized in RIPA buffer (1% NP-40, 1% sodium deoxycholate, 0.1% SDS, 150 mM NaCl, 25 mM TrisHCl, pH 7.6) and a cocktail of protease inhibitors (Complete, Roche, Indianapolis, IN). Protein concentration in extracts was determined using BCA reagent (Pierce Biotechnology, Rockford, IL). Equal amounts of protein were loaded and separated by SDS-PAGE in 4–12% NuPAGE Novex Bis-Tris Gels and NuPAGE MOPS SDS running buffer (Invitrogen, Carlsbad, CA) and then electro-transferred to 0.45-µm nitrocellulose membranes (Invitrogen, Carlsbad, CA). The membranes were probed with mouse monoclonal anti-REP-1 antibody, clone 2F1 (Santa Cruz Biotech, Santa Cruz, CA) generated against the 415 C-terminal amino acids of Rep1 [5], [27] and anti-REP-2 antibody C-20 (Santa Cruz Biotech, Santa Cruz, CA). Immunoreactivity was detected with a corresponding second anti-IgG antibody conjugated with horseradish peroxidase (Zymed Lab. Inc., San Francisco, CA) and imaged with X-ray film using SuperSignal West Pico Chemiluminescent Substrate (Pierce, Rockford, IL).

### Fluorescent Detection Using FACS Analysis

In brief, primary control and CHM monocyte or fibroblast cells were analyzed at the NIH Gene Therapy Flow Core using a FACSCanto (Becton Dickinson, San Jose, CA) equipped with a Coherent Sapphire 488 blue and JDS Uniphase HeNe Air Cooled 633 red laser. Cells containing pHrodo-TM Bioparticles-R, DQ-ovalbumin-BODYPY FL-dye or collagen-coated FluoSpheres were analyzed with BD FACSDiva software (Becton Dickinson, San Jose, CA). Appropriate gating of forward and side scatter excluded dead cells, unincorporated particles, and debris. Intensity of pHrodo fluorescence was measured with a 585/42 (564–606 nm) band pass emission filter and collagen-coated FluoSpheres and DQ-ovalbumin-BODYPY FL-dye were measured using a 530/30 (515–545 nm) band pass emission filter. Negative controls (unfed cells and no cells plus particles) were used to establish background and autofluorescence. For each sample, 5000 live-gated cells were acquired for all analyses. Positive (“shifted”) cells were gated and compared to the negative control.

### Microarray Data Collection and Normalization

The signal intensity for each of 54,675 probesets on the hybridized Affymetrix Human Genome U133 plus 2.0 chips was calculated using the MAS5 algorithm implemented in the GeneChip® Operating Software 1.4 (Affymetrix, Santa Clara, CA). Signal intensity values were normalized using an adaptive variance stabilizing, quantile-transformation named S10 (PJ Munson, GeneLogic Workshop of Low Level Analysis of Affymetrix GeneChip Data, 2001, software available at http://abs.cit.nih.gov/MSCLtoolbox). In order to apply statistical tests to the data, the variance of the replicates should be nearly uniform over the range of expression levels. The S10 transformation approximates a common logarithm transform over the central working range of the assay and thus differences of S10-transformed data values can be interpreted as log ratios or simply “log-fold changes”. A major advantage of this approach over the ordinary log-ratio is that changes in S10-transformed values have a uniform variance over the full expression scale. Data were collected under the MIAME compliant format and the raw data have been deposited into the Gene Expression Omnibus hosted by the NCBI (GEO; http://www.ncbi.nlm.nih.gov/geo/query/. With query accession no: GSE17549).

### Outlier Detection and Statistical Analysis

Visualization of the global relationships between the samples and detection of possible outliers among the 16 samples was facilitated by principal component analysis (PCA) of the S10 transformed data. PCA is a statistical technique used for representing high dimensional data in a few dimensions in order to visualize the relative location of the chips. Plotting the principal components containing the largest amount of variability against one another allows for outlier detection between the samples.

Because different cell types (monocytes and fibroblasts) and two distinct preparation techniques for monocytes were used, CHM and control samples were compared separately for each of three groups. To address variability between the samples a consistency test was used to determine significance of observed differences between CHM and control samples http://stat- http://stat-www.berkeley.edu/users/terry/zarray/Affy/GL_Workshop/genelogic2001.html [28], [29]. The false discovery rate (FDR) was calculated from the consistency test p-values [30].

### Biological Process Categorization by Gene Ontology

Functional annotation and classification, identification of significantly enriched biological themes using the Gene Ontology terms and classification of protein functional domains were explored in RPE gene signature list using The **D**atabase for **A**nnotation, **V**isualization and **I**ntegrated **D**iscovery (DAVID) bioinformatics resource (http://david.abcc.ncifcrf.gov/), and Expression Analysis Systematic Explorer (EASE) (http://apps1.niaid.nih.gov/david). Significant overrepresentation of functional classes of interest in the in RPE gene signature list was determined using GO terminology “biological process” (http://www.geneontology.org). This approach allows one to determine which particular functional categories are significantly overrepresented in the list of RPE specific gene. EASE score or Fisher's exact test p–value was used to measure significance of the gene-enrichment of the annotation terms within each biological process category.

## Results

### The Phenotype of CHM Patients

**Figure 2:**
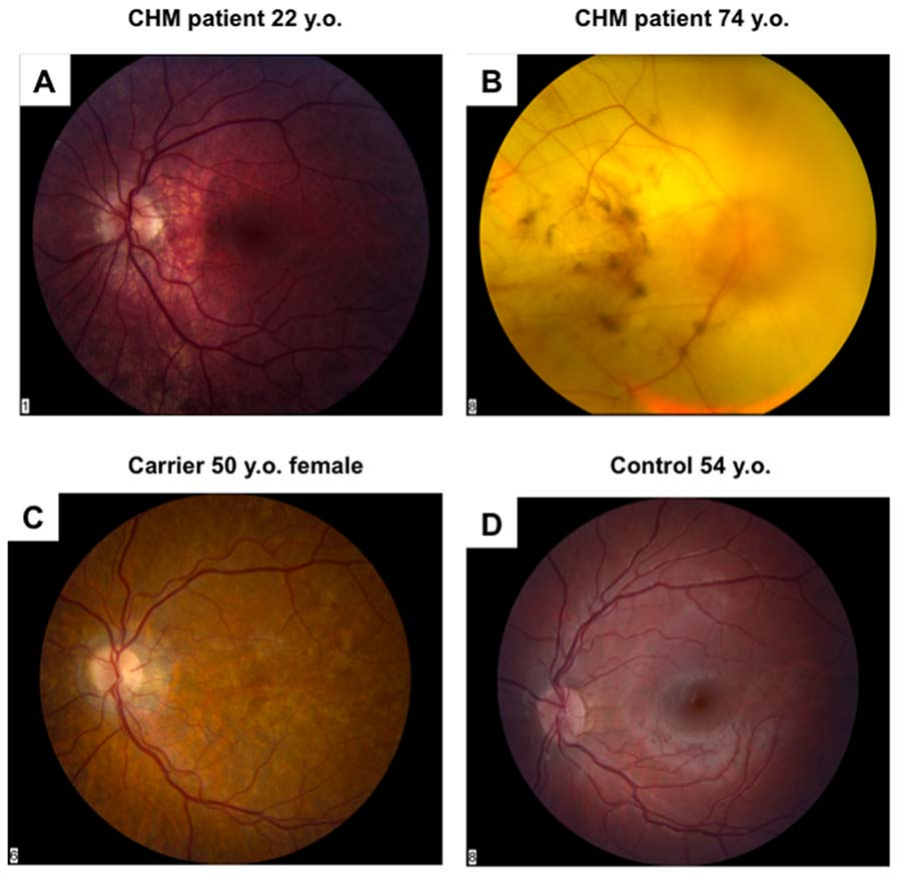
Fundus photographs of the control and CHM patients. **a.** CHM patient 22 y.o. characterized by RPE depigmentation and widespread RPE disruption **b.** CHM patient 74 y.o. characterized by loss of RPE and choroid, scattered pigment in macula, faint deep choroidal vessels and severely narrowed retinal vessels and optic nerve pallor (**Table 1**, CHM 9 and 10 respectively). **c.** Female CHM carrier, age 50 showing patchy RPE hypopigmentation without pigment dispersion and control subject. **d.** Fundus photograph of the normal eye.

Clinical data from CHM male patients are shown in the **Table 1**. Many males with CHM had mild to high myopia ranging from −0.50 to −10.625 (spherical equivalent, mean −2.83, n = 20 eyes). Retinal findings varied with the age of patients, with younger patients showing areas of moderate, widespread RPE atrophy (**Fig. 2A**). Older patients showed extensive choriocapillaris and RPE atrophy with preservation of central function and in later stages, retinal vessel attenuation and optic nerve pallor (**Fig. 2B**). The horizontal continuous visual field of patients showed a general decline with age. The visual field was estimated with the III4e target of Goldmann perimetry in patients ages 13 and 22 to be to 12–145 degrees (n = 8 eyes); at ages 29–49, 5–20 degrees (n = 10 eyes); and finally at ages 54–74, from no recordable field to 15 degrees (n = 8 eyes). The full field ERG of a patient at age 22 (CHM10) showed reduced scotopic and photopic a and b wave amplitudes and flicker responses. At age 29, the ERG of another patient (CHM1) showed severe reduction in the amplitudes of the scotopic b wave and photopic responses only 10% of normal. In 2 older patients, aged 49 (CHM4) and 54 (CHM5), the amplitudes of the photopic flicker ERGs measured approximately 2 µV (**Table 1**).

### The Genotype of CHM Patients

Molecular genetic testing of DNA from the CHM patients (CHM1–CHM11) revealed 9 different mutations located in exons 1, 5, 6, 7, 10, 13 and 14 (**Table 1**). Patient CHM2 had a full deletion of the *CHM* gene and CHM10 patient had no mutation in the coding sequences of the *CHM* gene or in adjacent splice sites. This patient had clinical features of CHM (**Fig. 2A**); real time PCR analysis failed to show copy number variants of any of the exons of the *CHM* gene, but protein analysis confirmed absence of REP-1 (data not shown). The mutation in this patient is possibly localized within an intron or the 5-prime region of the gene.

**Figure 3:**
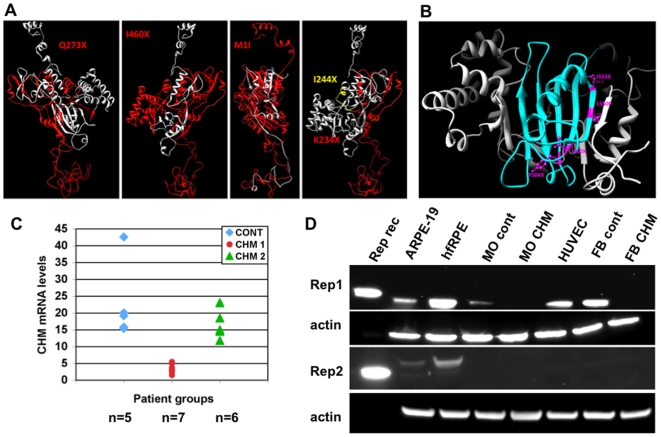
Effect of different mutations on the structure and levels of REP-1 mRNA and protein. **a**, Effect of different nonsense mutations on the structure of REP-1 protein (Q273X, I460X, M1I and K234X). **b**, Distribution and position of the mutations in the REP-1 protein, note that 4 of 9 mutations localized in the beta sheet of the REP-1 (blue) and 7 of 9 mutations (P179X, K234X, I244X, I460X, Y504 X, L550P and I553X, **Table 1**) localized to domain 2 of the REP-1 protein. **c**. Levels of mRNA determined by the microarray analysis of the expression profiles from monocytes and fibroblasts from CHM and control patients. Control group, n = 5; group CHM1 includes patients with low levels of REP1 mRNA, n = 7; group CHM2 includes patients with REP-1 mRNA similar to the controls, n = 6. **d**. Expression levels of REP-1 and REP-2 in different cell types derived from CHM and control patients. Lane: 1, 10 ng of rat recombinant REP-1 or 10 ng of rat recombinant REP-2 with HisTag; cell lysates (40 µg of protein for each) 2, ARPE19; 3, human fetal RPE; 4, MO- monocytes from control; 5, MO-monocytes from patient CHM4; 6, cultured human umbilical vein endothelial cells (HUVECs); 7, primary fibroblasts from control; 8, primary fibroblasts from CHM2 patient. β-actin was used as a loading control.

For each of the other 9 mutations, the predicted size of the protein product and the structural change in REP-1 are presented in **Table 1**. Five mutations are newly described in this study: c.1327_1328delAT (p.I460X), c.817C>T (p.Q273X), deletion of exons 6 and 7 (p.I244X), c.700A>T (p.K234X) and p.M1I; and 4 mutations were structurally characterized previously: c.525_526delAG (p.P179X); c.1542T>A, STOP (p.Y504X) and c.1646delC (p.I553X) and p.L550P (Sergeev et al, 2009). The effect of mutations K234X, I244X, Q273X and I460X on REP-1 structure is demonstrated in **Fig. 3A**. The truncated parts of the REP-1 molecule are shown in red. All nonsense mutations result in a truncated protein product with unstable protein structure and loss of critical REP-1 function. The missense mutation M1I (**Table 1**) occurs at the start site (ATG) resulting in a truncated protein product (white ribbon, **Fig. 3B**) with the loss of REP-1 domain structure. Molecular modeling of the missense mutation, L550P, predicted that the structure of the protein is unstable which could be one of the mechanisms of REP-1 loss [22]. Western Blot analysis confirmed absence of the REP-1 protein in the fibroblasts from all patients carrying these mutations (**Fig. 3D**).

### REP-1 and REP-2 Expression

Microarray and quantitative PCR analysis confirmed that fibroblasts and monocytes from CHM patients with different mutations express *CHM* mRNA at different levels. CHM subjects 1, 5, 7, 8, and 11 (CHM group 2) express *CHM* mRNA at levels similar to controls but the levels of *CHM* mRNA in CHM subjects 2, 4, 6 and 9 (CHM group 1) were 8–20 times lower (**Fig. 3C**). Similar CHM mRNA levels were obtained for monocytes and fibroblasts derived from the same patient. This result could be explained by the nature and position of the mutations, and their effect on the structure of mRNA. In those with premature termination codons, the mRNA could potentially be targeted for nonsense-mediated decay; while in others, the mutations could affect the stability of the mRNA [31].

Levels of REP-1 protein expression varied in different cell types, with less expression in monocytes and ARPE19 cells compared to human fetal RPE cells, fibroblasts or vascular endothelial cells (**Fig. 3D**). We determined that the levels of REP-1 and REP-2 expression are quite variable between different human tissues. We also observed a lack of apparent correlation between the REP-1 and REP-2 levels in these cells, which could indicate another compensatory mechanisms employed by the peripheral cells in CHM patients.

### Phagocytosis in Monocytes of CHM Patients and Controls

**Figure 4:**
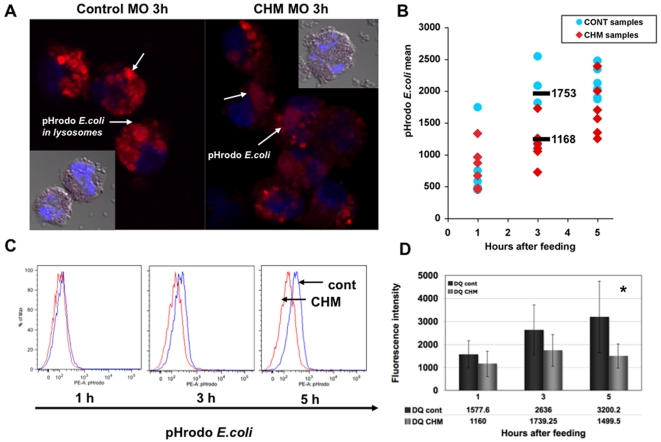
Levels of lysosomal acidification and proteolytic degradation in monocytes from CHM patients and age-matched controls. **a.** Intralysosomal acidification measurements were performed using *E. coli* BioParticles conjugated with pH dependent dye (pHrodo). Representative confocal images of the monocytes from patient CHM8 (right panel) and age matched control (left panel) feed with BioParticles at 3 h following the feeding. Acidic environment of the lysosomes caused an increase in fluorescent intensity of the BioParticles engulfed by the cells (in red, blue is a DAPI nuclear staining). **b.** Levels of fluorescence of the monocytes from CHM (n = 7) and control (n = 6) patients feed with BioParticles were analysed by flow cytometry, data expressed as a mean fluorescence intensity+/−SED at 1, 3 and 5 h following the feeding. Fluorescence intensity of pH dependent BioParticles taken up by monocytes from CHM patients was lower compared to the control at each time point. **c.** Representative FACS histograms showing shift in fluorescence intensity between the CHM and control monocytes fed with *E. coli* pHrodo at 1, 3 and 5 h. **d.** The efficiency of lysosome-mediated proteolytic degradation by monocytes from CHM (n = 7) and control (n = 6) patients assessed with DQ-ovalbumin particles. DQ-ovalbumin is a self-quenched substrate for proteases, which becomes fluorescent after proteolytic cleavage. Increase in fluorescence intensity, corresponding to the rate of proteolytic degradation of DQ-ovalbumin, was measured by flow cytometry. Data was expressed as a mean fluorescence intensity of the cells+/−SED at 1, 3 and 5 h following the feeding. Rates of proteolytic degradation were significantly lower in monocytes from CHM patients compared to control (• p = 0.005).

*E. coli* particles conjugated with a pH-dependent fluorescent dye (pHrodo™ BioParticles®) were used to track the phagocytic process in monocytes from CHM patients and healthy age-matched controls. In the neutral pH outside and inside the cell, the light emission by pHrodo conjugated *E. coli* is dim and barely detectable by the fluorescent microscope or flow cytometry analysis (FACS). Once BioParticles are engulfed, the acidic environment within the phagolysosome causes a significant increase in fluorescent emission by the particles. The use of pH-dependent BioParticles allowed us to determine the number of phagocytosing cells in the population, the kinetics of uptake and change in pH of the lysosomes. Monocytes from 8 CHM patients with different *CHM* mutations and 6 age-matched controls were compared over time for different aspects of phagocytosis and intracellular vesicle transport using live cell imaging and FACS. Cells from CHM subjects were analyzed in parallel with appropriate age-matched controls and results were reproduced in 6 independent experiments. FACS and confocal microscopy at 1 h, 3 h and 5 h following the feeding confirmed the increase in fluorescent intensity of BioParticles engulfed by monocytes (**Fig. 4A, C**). Dramatic increase in fluorescence intensity was observed for 67–89% of the control and patients monocytes one hour after the feeding with *E. coli* pHrodo. Confocal imaging of the phagocytosing CHM and control monocytes demonstrated the presence of fluorescent BioParticles inside of the phagolysosomes (**Fig. 4A right panel**). FACS analysis was done on the large population of phagocytosing monocytes from 7 CHM and 6 controls to determine the differences in fluorescence intensity of the cells following the feeding (**Fig. 4B, C**). Flow cytometry analysis showed that CHM monocytes uptaking *E. coli* pHrodo demonstrated consistently lower levels of fluorescence compared to controls. The biggest difference in fluorescence levels was observed by 3 h following feeding with the BioParticles (1168±345 in CHM monocytes, 1753±453 in controls) (**Fig. 4B**). These results are consistent with the hypothesis that phagolysosomes in monocytes from CHM patients do not develop the same level of acidification as control monocytes resulting in a relative decrease in fluorescence levels. The increase in pH level of the phagolysosomes could affect the ability of the cells to adequately process and degrade engulfed material.

The efficiency of lysosome-mediated degradation by monocytes was further evaluated with DQ™ ovalbumin particles with BODYPY FL dye. DQ-ovalbumin is a self-quenched substrate for proteases, which becomes fluorescent after proteolytic cleavage. Using this system, we evaluated the effect of increased acidification on proteolysis in lysosomes of monocytes from CHM patients and controls. FACS analysis of the monocytes from 7 CHM patients and 6 controls following feeding with DQ-ovalbumin particles confirmed lower levels of fluorescence in CHM patients, which reflected decreased rates of DQ-ovalbumin degradation by the cells. Higher levels of fluorescence were observed in control cells at 3 h following feeding (1739±696 in CHM monocytes, 2636±1084 in controls) and at 5 h the difference became statistically significant (1499±526 in CHM monocytes, 3200±1548 in controls; p<0.05) (**Fig. 4D**). Some patients displayed a certain degree of the variability in the fluorescence intensity that may be due to the potential residual activity of some mutant forms of REP-1.

### Level of Acidification and Rate of Degradation of DQ-Ovalbumin Affected in CHM and Control Monocytes after the Treatment with Bafilomycin A1 (BafA1)

**Figure 5:**
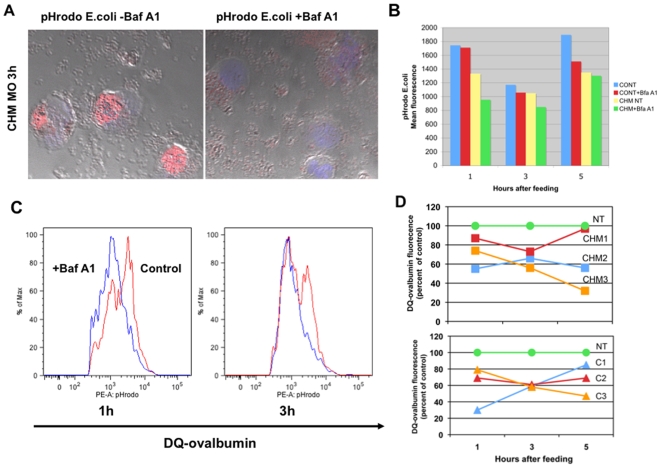
Lysosomal acidification and rate of proteolytic degradation in monocytes from CHM and control patients treated with Bafilomycin-A1 (BafA1). **a.** Lysosomal acidification and rate of proteolytic degradation in monocytes from CHM and control BafA1. Intralysosomal acidification measurements were performed using *E. coli* BioParticles conjugated with a pH dependent dye (pHrodo). Treatment caused an increase in lysosomal pH as evident by a decrease in the fluorescence of BioParticles (confocal images, left panel vehicle no effect, right panel cells pre- treated with BafA1 for 30 min, decreased fluorescence). **b.** Decrease in fluorescence levels of BioParticles following the treatment with BafA1 in monocytes from control and patient CHM4 measured by flow cytometry analysis at 1, 3 and 5 hours following the feeding. **c.** Representative FACS histograms showing a shift in fluorescence intensity of the CHM and control monocytes fed with BioParticles treated with BafA1 at 1, 3 and 5 h. **d.** Decreased rate of DQ-ovalbumin degradation in CHM (n = 3) and control (n = 3) patients before and after the treatment with BafA1 measured by flow cytometry analysis at 1, 3 and 5 hours following the feeding. Data expressed as a percent of fluorescence reduction in CHM and control cells treated with BafA1, compared to the non-treated (NT) cells.

To confirm that the increase in lysosomal pH affected the ability of monocytes to degrade engulfed material, cells from controls and CHM patients were treated with the vH+ATPase inhibitor, bafilomycin A1. A number of studies have demonstrated that bafilomycin A1 causes an increase in pH inside the lysosomes, leading to a decrease in the rate of degradation of material in lysosomes [32]. Change in the acidification of lysosomal environment of monocytes was measured by the change in fluorescence of pH-dependent BioParticles (**Fig. 5A–C**) and the rate of proteolytic degradation in lysosomes was measured using DQ-ovalbumin. CHM and control monocytes were pretreated with 100 nM bafilomycin A1 for 30 min before the feeding. FACS analysis of the monocyte population from CHM patients (n = 3) and controls (n = 3) at 1, 3 and 5 h following the treatment with bafilomycin A1 demonstrated a decrease in the levels of fluorescent intensity of engulfed pH-dependent BioParticles in both controls and patients. CHM and control lysosomes demonstrated a decrease of 7–47% in fluorescence intensity depending on the time point. The fluorescence level for a selected patient before and after bafilomycin A1 treatment is shown in **Fig. 5B**. Corresponding rates of DQ-ovalbumin degradation were also decreased in both CHM and control monocytes 1 and 3 h following pre-treatment with bafilomycin A1 (**Fig. 5D**). These results confirm that decreased acidification in lysosomes affects the proteolytic abilities of CHM and control monocytes.

### Trafficking Defects in Primary Skin Fibroblasts (FB) from CHM Patients

**Figure 6:**
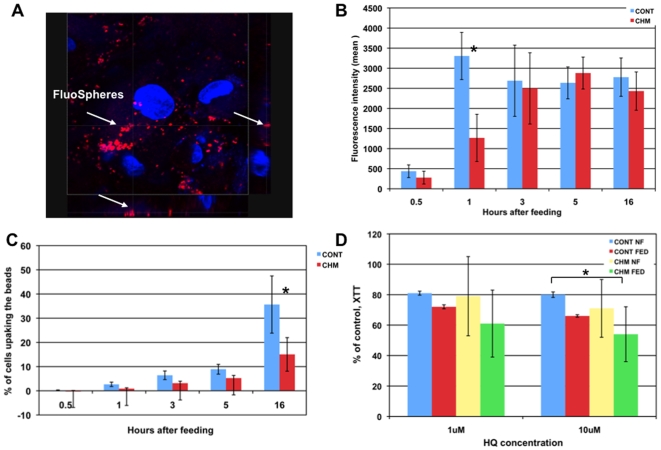
Differential uptake of collagen coated beads (FluoSphere) by primary fibroblasts from CHM and control patients. **a.** Confocal image of the fibroblasts 1 h after the uptake of collagen coated fluorescent beads. Note that beads were internalized into the cytoplasm. **b.** Rates of bead uptake 1 h following feeding was significantly higher in the control cells (n = 7) compared to the CHM patients (n = 6) as measured by flow cytometry. Data expressed as mean fluorescence intensity+/−SED at 1, 3, 5 and 16 h. After that CHM cells demonstrated a similar rate of the uptake. **c.** Percent of cells taking up beads was consistently higher in the control cells (16 h period). **d.** CHM fibroblasts (n = 5) cultures fed with beads less resistant to the oxidative stress initiated by the treatment with 1 µM and 10 µM of hydroquinone (HQ) when compared to controls (n = 3). Viability levels were measured using XTT and expressed as percent viability of non-treated non-fed cells.

Primary dermal fibroblasts from 7 CHM patients and 6 controls were used to determine if trafficking defects observed in monocytes from the CHM patients were present in other peripheral cells of the patients. Phagocytosis in primary fibroblasts was tracked and quantified using collagen I coated FluoSpheres beads. The rate of uptake was significantly lower in fibroblasts from CHM patients compared to controls 1 h following feeding; after that, CHM patients demonstrated similar rates of uptake (**Fig. 6B**). The number of cells taking up particles was consistently smaller over the 16 h time period in primary cultures of CHM patients (**Fig. 6C**). CHM fibroblasts cultures fed with beads were also less resistant to the oxidative stress initiated by treatment with 1 µM and 10 µM of hydroquinone (HQ) (**Fig. 6D**). These data imply that primary fibroblasts from CHM patients exhibit a number of defects in the trafficking system when compared to the control cells.

### Microarray Analysis of Expression Profiles of CHM and Control Patients Derived from Monocytes and Fibroblasts

**Figure 7:**
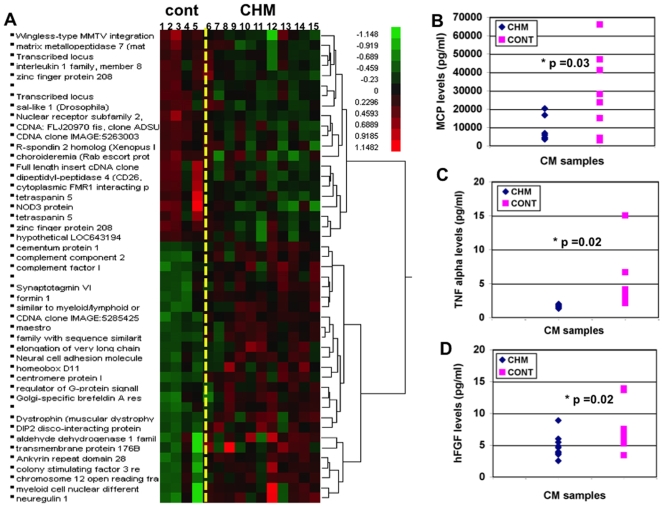
Mutation in REP-1 affects gene expression and secretion in CHM patients. a. Hierarchical cluster of 47 probe sets in control and CHM samples. Using consistency testing, twenty-six probe sets were found to be significantly over-expressed and 21 under-expressed in monocytes and primary fibroblasts cells from CHM patients 43 (CHM 6-15) compared to control (Cont 1-5)(p<0.0001, FDR30%) b-d. Level of secretion of the cytokines and growth factors by primary fibroblast cultures into conditioned media. MCP-1, TNF-alpha and FGF factors were detected at significantly higher levels in samples collected from the control cells (n = 9) compared to conditioned media samples from 8 CHM patients (p<0.005)

PCA and hierarchical cluster analysis were performed on expression profiles of 16 samples to evaluate the grouping between the primary fibroblasts cultures and monocytes from CHM and control patients. Differentially expressed probesets were determined from the comparison of 11 CHM patients' monocytes and primary fibroblasts to those of 5 control subjects using a consistency test [28], [29]. The dendrogram of the hierarchical clusters of twenty-six probe sets that are significantly over-expressed and twenty-one probe sets that are under-expressed in CHM patients is shown on the Figure 7A (p<0.0001, FDR 30%). The **D**atabase for **A**nnotation, **V**isualization and **I**ntegrated **D**iscovery tool (**DAVID**) was used to carry out a functional classification and annotation and ascertain the enrichment of particular biological gene ontology categories (GO)[33]. Functional categories affected in both fibroblasts and monocytes of CHM patients included a large group of genes involved in regulation of cell adhesion and motility (NCAM-2, FMN1, ANKRD28, CSF3R, DMD, MLLT4, DPP4, TSPAN5, CD114, DMD, CYFIP2, WNT5A, MMP7), immune response (IL1, CC2, CFI, ELOVL2), regulation of trafficking and exocytosis (RGS11, SYT 6, GBF1) and transcriptional regulation (HOXD11, MRO, NRG1, NLRC3, NR2F6, SALL1, DIP2A, RSOPO2) (**Fig. 7A**). Interestingly, expression levels of genes encoding secreted proteins from different functional groups including CFI, CC2, NRG1, MMP7, WNT5A, RSOPO2, CSF3R, DPP4, IL1 were deregulated altered in CHM cells compared to the control. As shown before, impairment of decreased acidification in lysosomes and proteolytic degradation could affect exocytosis and secretion processes by the cells. We hypothesized that cells derived from the CHM patients could have a general impairment in the mechanism of exocytosis.

### Levels of Cytokine and Growth Factors Released by the Primary Fibroblast Cultures Derived from the CHM Patients

To determine the effect of mutant REP-1 on secretion of cytokines and growth factors by the peripheral cells, conditioned media samples were collected from the primary fibroblast cultures of 8 CHM patients and 9 controls. Levels of IL-8, MCP-1, TNF-alpha, FGF and PEDF factors were significantly higher in samples collected from the control cells (p<0.005), and levels of VEGF and EGF did not change significantly. The results were confirmed in 3 independent experiments. Primary fibroblasts from CHM patients released 3.2 fold less IL8, 4 fold less MCP1, 2.8 fold less TNF-alpha, 2.33 fold less FGF and 1.6 fold less of PEDF. Actual levels of secretion of MCP-1, TNF-alpha and FGF for all the controls and CHM samples are presented in **Fig. 7B, C and D**. Reduced levels of secretion of multiple factors corroborate the results of the microarray analysis and point to the possible impairment of the secretion process by cells expressing mutant REP-1.

## Discussion

CHM is a monogenic disease caused by various mutations in the *CHM* gene that result in the loss of function of Rab escort protein (REP-1) and cause slow degeneration of RPE, choroid and photoreceptors. While many genetic studies have characterized the types of mutations in REP-1 in CHM patients, few have added to our understanding of the pathogenesis of the disease. REP-1 is involved in the prenylation of Rabs (small Ras superfamily GTPases), which are essential for phagocytosis, secretion and intracellular trafficking in variety of tissues throughout the body [1], [27], [34]. A *CHM-like* gene in mammals encodes REP-2, which is thought to partially compensate for the lack of REP-1 in all tissues except the eye in CHM patients [10]. Normally REP-1 is expressed in every cell type in the body so it is not clear why the absence of REP-1 affects only the retina. One suggestion was that certain Rabs are prenylated more efficiently by REP-1 than REP-2 [35]. For example, Rab27 is under-prenylated in lymphoblasts from the CHM patients and probably preferentially requires REP-1 [8]. Rab27 is also present at high levels in the RPE and choriocapillaris and its under-prenylation could contribute to the degenerative process. To study phagocytosis, secretion and intracellular trafficking in CHM patients, we are limited by the lack of functionally relevant cell culture models pertinent to the eye, which are functionally deficient in REP-1. For example, human RPE cultures models would be hard to obtain from CHM patients, and further culture and manipulate. The use of peripheral cells and primary cultures derived from CHM patients could be extremely helpful since the existing paradigm for CHM pathogenesis is lacking a number of key data regarding the tissue specificity of REP-1 regulation. In addition, peripheral cells and primary cultures were derived from CHM patients with variety of mutations could help to confirm if different mutations cause similar phenotypes. This approach has allowed us to overcome the limitations of animal models and directly evaluate disease phenotype in different tissues. To our knowledge, this is the first study to look at the effect of different mutations in REP-1 on phagocytosis, trafficking and proteolytic degradation in peripheral tissues of CHM patients.

### Phagocytosis and Proteolytic Degradation Is Altered in Monocytes from CHM Patients

Monocytes from CHM patients demonstrated a consistent increase in lysosomal pH when compared to controls. Since an increase in lysosomal pH leads to the inhibition of lysosomal hydrolases, this could dramatically affect the ability of these cells to process and degrade engulfed material [32], [36]. Indeed, using a self-quenched DQ-ovalbumin particle system, we confirmed that the decreased acidification of lysosomes of CHM patients significantly affected proteolytic abilities of the monocytes [25], [26]. Monocytes from different CHM patients demonstrated significant variation in the rates of uptake of material, lysosomal acidification and protein degradation, which could be explained by the effects of different mutations in the *CHM* gene on structure and function of REP-1. Monocytes from CHM patients showed the same functional defects when compared to controls.

The RPE serves a number of vital functions in the eye including daily phagocytosis and degradation of shed photoreceptor outer segments; maintenance of the visual cycle by the uptake, processing, and transport of vitamin A; and the transport of nutrients between the choroid and the RPE [37], [38], [39], [40]. Dysfunctional lysosomal processing in the RPE could contribute to the pathophysiological events that cause RPE damage and degeneration of the neurosensory retina [41], [42]. Bergmann and coworkers demonstrated that A2E, which accumulates in age-related macular degeneration (AMD), impaired the proton pump of the lysosomal membrane in cultured RPE, altered the function of the lysosome and thereby slowed the processing of photoreceptor outer segments [36]. Impaired lysosomal function and protein degradation abilities in RPE likely play a significant role in the development of AMD and also chloroquine-induced retinal degeneration [17], [43], [44], [45], [36]. Chloroquine accumulation within the retina increases lysosomal pH, reduces the activity of lysosomal enzymes and leads to the accumulation of undegraded oxidized residual material [41]. Further, inhibition of lysosomal enzymes in the RPE leads to attenuation of exocytosis and release of incompletely degraded phagolysosomes into the basal and intercellular spaces between adjacent cells [17], [20], [46]. Post-mitotic cells like the RPE, with an extended lifetime and low turnover *in vivo*, may be especially susceptible to toxicity and degeneration caused by defects in lysosome-mediated degradation and impaired secretion. The RPE and photoreceptors in a chronic disorder such as CHM are probably constantly subjected to the stress caused by the lysosomal dysfunction and accumulate sub-toxic changes with age, which lead to the development of disease. We observed, for example, that fibroblasts of CHM subjects were less resistant than controls to oxidative stress induced by hydroxyquinone, and could suggest that the cells of the RPE of these subjects are similarly less resistant to oxidative stress than normal subjects.

The altered cellular phenotype of fibroblasts and monocytes from CHM patients carrying knockouts and missense mutations of the CHM gene complement and expand the existing data resulting from knock down of the main transcript of the CHM gene in cultured human fetal RPE cells [47]. The siRNA approach on cultured RPE cells had a number of inherent limitations that prevented extrapolation and generalization of findings to human subjects. First, the mock, non-target siRNA procedure by itself caused an increase in the pH of the lysosomes for an extended period of time and therefore the resulting effect on the rates of proteolytic degradation within the RPE could be interpreted only in a hypothetical sense. Secondly, the CHM siRNA resulted in only a 70% reduction in the level of expression of REP-1. Measurable levels of REP-1 were still present, unlike that of the cells from CHM patients, which in general lack REP-1. Finally, the effect of the proprietary siRNA mixture was not validated for splicing variants that are known for the CHM gene transcript (two major and several tissue-specific ones). Therefore, by studying naturally occurring mutations in the present work we were able to overcome these limitations and address the pathogenesis of CHM directly. Using this approach we clearly demonstrated for the fist time, that levels of acidification in phagolysosomes are increased in peripheral cells of CHM patients leading to the significantly reduced rates of proteolytic degradation. Therefore, data obtained from the RPE REP-1 siRNA model and contrasted with that obtained from the study of peripheral cells of the CHM patients reveal valuable and novel information with respect to understanding the mechanism and development of the CHM disease in the human population.

### Exocytosis and Secretion Affected in Peripheral Cell from CHM Patients

A number of genes involved in the immune response, small GTPase regulation, secretion, the regulation of transcription, cell adhesion and the regulation of exocytosis were significantly up-and down-regulated in both fibroblasts and monocytes from CHM patients. Under-prenylation of certain Rabs could alter exocytic pathways and secretion in a variety of cells [35]. Previous studies have suggested that the reduction or absence of REP1 could have a variable effect on the prenylation of Rabs and therefore could affect different aspects of the trafficking in the RPE and other cell types [7], [48], [12]

We hypothesized that underprenylation of certain Rabs would probably influence the pH level in lysosomes, and exocytosis/secretion of cells. Indeed knock down of the CHM gene resulted in an increase in the secretion of monocyte chemotactic protein-1 (MCP-1) and interleukin-8 (IL-8) by human fetal RPE cells [47]. These cells, while maintaining a polarity in culture, secreted more MCP-1 and IL-8 to both apical and basal compartments in culture. In contrast, fibroblasts from CHM patients, when compared to normal controls, secreted significantly lower levels of MCP-1, PEDF, TNF-alpha, FGF and IL-8. Differences observed between the secretion patterns of the human fetal RPE after REP-1 knock down and peripheral cells from CHM patients could be explained by: (1) the differences in physiology of adult and fetal tissues, (2) the difference in prenylation levels of specific Rab proteins in RPE and other cell types leading to differences in the regulation of secretion in different tissues. These effects of relative absence of REP-1 and absence of REP-1 on secretion have not been previously reported. Differences in the regulation of secretion between the different cell types with reduced/absent levels of REP-1 could explain the tissue specific degeneration that occurs in choroideremia. For example, Rab27a plays a crucial role in the exocytosis of lytic granules in cytotoxic T lymphocytes and Rab27b is a key regulator of dense granule secretion in platelets and other secretary cells [18], [49], [50], [51], [52]. Rab27 is also present at high levels in the RPE and choriocapillaris and its under-prenylation could contribute to the degenerative process. Interestingly, whereas Rab27 is also expressed in a variety of secretary and neuronal cells involved in synaptic transmission [19], [53], [54], [55], [56]; neuronal degeneration, outside the retina, has not been reported in CHM patients. Increased exocytic activity of damaged RPE cells has been proposed to play a role in the development of AMD and the retinal degeneration due to the chloroquine toxicity. Possibly the disordered cell function observed in fibroblasts and monocytes from CHM patients represents a subclinical CHM phenotype that was not previously recognized and offers the opportunity for further research.

### Different Mutations in CHM Gene Could Affect mRNA and Protein Stability

Mutated forms of REP-1 (**Table 1**), except L550P, were all visibly absent in primary fibroblasts and monocytes derived from CHM patients. Of interest, 4 mutations in the *CHM* gene (I460X, Y504 X, L550P and I553X) occur in domain 2 (residues 93–270 and 447–553), almost the same area of the β-sheet formed by residues 546–556, 450–460, 500–515 shown in cyan (**Fig. 3A, B**). In addition, 7 mutations (P179X, K234X, I244X, I460X, Y504X, L550P and I553X, **Table 1**) localized to the same domain.

While it has been suggested that all the mutations in CHM patients lead to the loss of the REP-1, the mechanism of the loss could range from nonsense-mediated decay of the coding mRNA to the degradation of the truncated/misfolded REP-1 in the endoplasmic reticulum. The latter mechanism could cause significant stress and toxicity to the cell and eventually lead to degeneration and cell death [57], [58], [59] In support of that, we observed that a missense mutation (L550P), which does not change the length of the protein significantly affects the level of REP-1 in cells in fibroblasts of the patient [22]. We hypothesized that the position of the mutation could significantly influence the stability of REP-1 and the severity of the CHM phenotype. Since the CHM RPE cells are not available for study, fibroblasts and monocytes from patients are the only viable models of CHM that allow genotype/phenotype correlations to be drawn; correlating individual mutations with cellular defects and the clinical severity of the disease. [60], [61].

Surprisingly, we did not see a compensatory increase in the protein and RNA levels of REP-2 in fibroblasts or monocytes from CHM patients. REP-2 expression was uniformly found to be very low. In addition, we observed a significant difference (more than 10 fold) in the levels of REP-1 mRNA in peripheral tissues of patients carrying different CHM mutations. Some patients' cells demonstrated REP-1 mRNA levels similar to control; however, in others the mRNA levels were quite low. Generally, low levels of CHM mRNA could be explained by nonsense-mediated decay of the transcript [31]. In our study, differences in mRNA levels between CHM patients could be determined by the position of mutations in REP-1 that may or may not target the mRNA for nonsense-mediated decay. This observation could lead to treatment strategies for patients, including chaperone molecules to prevent RNA degradation or small molecules that force the read through of premature stop codons in the *CHM* gene.

To date, no clinical studies have reported the effect of different mutations in the *CHM* gene on tissues other than the eye. Our observations indicate that despite the variety of mutations found in CHM patients, in each case the effect of the mutation was the same in disordering trafficking and exocytosis in fibroblasts and monocytes. As well, the retinal phenotype of these patients was typical of the spectrum expected at the various ages of the patients. The peripheral tissues of CHM patients will be useful models that reflect the defects in trafficking that underlie this retinal phenotype and provide additional data to explain the mechanism REP-1 compensation.
